# Prevalence of bovine trypanosomosis and tsetse fly density in the Yem special district: a cross-sectional study

**DOI:** 10.3389/fvets.2024.1460650

**Published:** 2024-12-23

**Authors:** Dagim Bekele, XiaoLong Wang, Ahimedin Beshir, Endale Terefe

**Affiliations:** ^1^College of Wildlife and Protected Area, Northeast Forestry University, Harbin, China; ^2^Key Laboratory of Wildlife Diseases and Biosecurity Management of Heilongjiang Province, Harbin, China; ^3^Animal Health Institute, Kality Animal Health Institute Research Center, Addis Ababa, Ethiopia; ^4^Animal Health Institute, Bedele Animal Health Research Center, Bedele, Ethiopia; ^5^Bedele District Livestock Development and Health Office, Bedele, Ethiopia

**Keywords:** bovine, prevalence, PCV, trypanosomosis, tsetse fly, Yem special district

## Abstract

This study assesses the prevalence of bovine trypanosomes and the density of tsetse flies in the Yem Special District, Southern Ethiopia, highlighting the disease's significant impact on livestock health and agricultural productivity. Conducted between May 2022 and January 2023, the cross-sectional survey analyzed 960 blood samples for trypanosomes prevalence and tsetse fly density. Results revealed a 10.63% (9–12%) overall prevalence of bovine trypanosomes, with *Trypanosoma congolense* (5.83%) and *Trypanosoma vivax* (4.73%) as the predominant species, and 0.63% of mixed infection. Significant associations were found between trypanosomes and anemia, age, and sex, with higher prevalence rates observed in cattle with poor body condition scores and black-hair coats. The study identified three Glossina species, with *Glossina morsitans submorsitans* being the most prevalent. The findings underscore the need for integrated vector control strategies, community engagement in disease management, and further research using molecular techniques for deeper understanding and effective control measures. Collaboration among stakeholders is crucial for mitigating the disease's impact on livestock and human populations.

## Introduction

Bovine trypanosomes, a parasitic disease caused by the protozoan Trypanosoma, is a significant barrier to cattle productivity, particularly in sub-Saharan Africa where it is widespread. This disease endangers the survival and productivity of cattle, affecting not only agricultural progress but also posing a risk to human health as one of the notable parasitic blood diseases (1, 76). The tsetse fly, belonging to the genus Glossina, serves as the primary vector, transmitting the most pathogenic Trypanosoma species in Ethiopia, including *Trypanosoma congolense, T. vivax*, and *T. brucei* (2, 75).

In Ethiopia, an estimated 180,000–220,000 km^2^ of land is tsetse-infested, putting ~14 million cattle at risk (2). The distribution of Trypanosoma species is confined mainly to the country's tsetse belt, which spans the western, southwestern, and southern regions, though *T. vivax* is capable of causing disease beyond this area (3, 75). Five Glossina species are present in Ethiopia, with four *G. morsitans submorsitans, G. pallidipes, G. tachinoides*, and *G. fuscipes fuscipes* being of significant economic concern. Mechanical transmission by other flies like Stomoxys and Tabanus has also been documented (4, 77).

The disease's impact extends beyond direct losses in meat and milk productivity, abortion, and mortality rates; it also has profound indirect effects on agriculture and livestock production, contributing to a national economic loss of ~200 million US dollars. The reduced efficiency of oxen for plowing and the challenge of introducing drought-resistant cattle breeds in tsetse-infested regions hinder agricultural development (5, 6). Addressing trypanosomes necessitates reducing the contact between cattle and vectors. Control strategies primarily include managing tsetse populations, administering trypanocidal drugs, and employing livestock breeds with disease tolerance (7, 8). A comprehensive understanding of the disease's epidemiology and vector distribution is crucial for devising effective interventions.

Multiple regional studies have been conducted on trypanosomes and the density of tsetse flies in different parts of Ethiopia. However, in the Yem special district, there is a notable limitation in the available research to comprehensively understand the prevalence of various trypanosoma species and the density of tsetse flies. The area's biodiversity includes various wild animals like African buffalos, bush pigs, warthogs, bushbucks, kudus, hippopotamuses, crocodiles, hyenas, and snakes, which are potential food sources for the vectors of the Trypanosoma parasite (9). Agriculture serves as the cornerstone of livelihoods in the study area, characterized by a mixed farming system where livestock plays a vital role. The livestock market activities, closely linked with neighboring areas such as Natiri, Kunbi, Deneba, and Sekoru, are significant contributors to the local economy.

Regional governments, NGOs, and international organizations like the International Livestock Research Institute (ILRI) collaborated in protecting livestock against trypanosomes and tsetse flies. But it was not sustainable. To address sustainability concerns, the Bedele Animal Health Research Center is now working on a solution. The local health infrastructure in the Yem special district, particularly in highly tsetse and trypanosomes hotspot areas, is inefficient due to challenges such as difficult land topography, limited logistics, potential wildlife reservoirs, lack of high technology diagnostic tools, and capacity building (10).

This lack of detailed information hinders the ability to inform the necessary control measures for this district. The problem statement for this study was lacking specific data regarding the prevalence of bovine trypanosomosis and the density of tsetse flies in the Yem special district. This knowledge gap significantly impedes the development and implementation of effective control measures and management strategies to mitigate the impact of this disease on the local livestock populations, which are vital to the livelihoods and food security of the Yem community.

This study aims to ascertain the prevalence and risk factors associated with bovine trypanosomes in the Yem special district and to evaluate the distribution and density of tsetse flies, which are pivotal for informing targeted control measures and promoting sustainable agricultural and livestock development.

## Materials and methods

### Study areas

**Figure 1:**
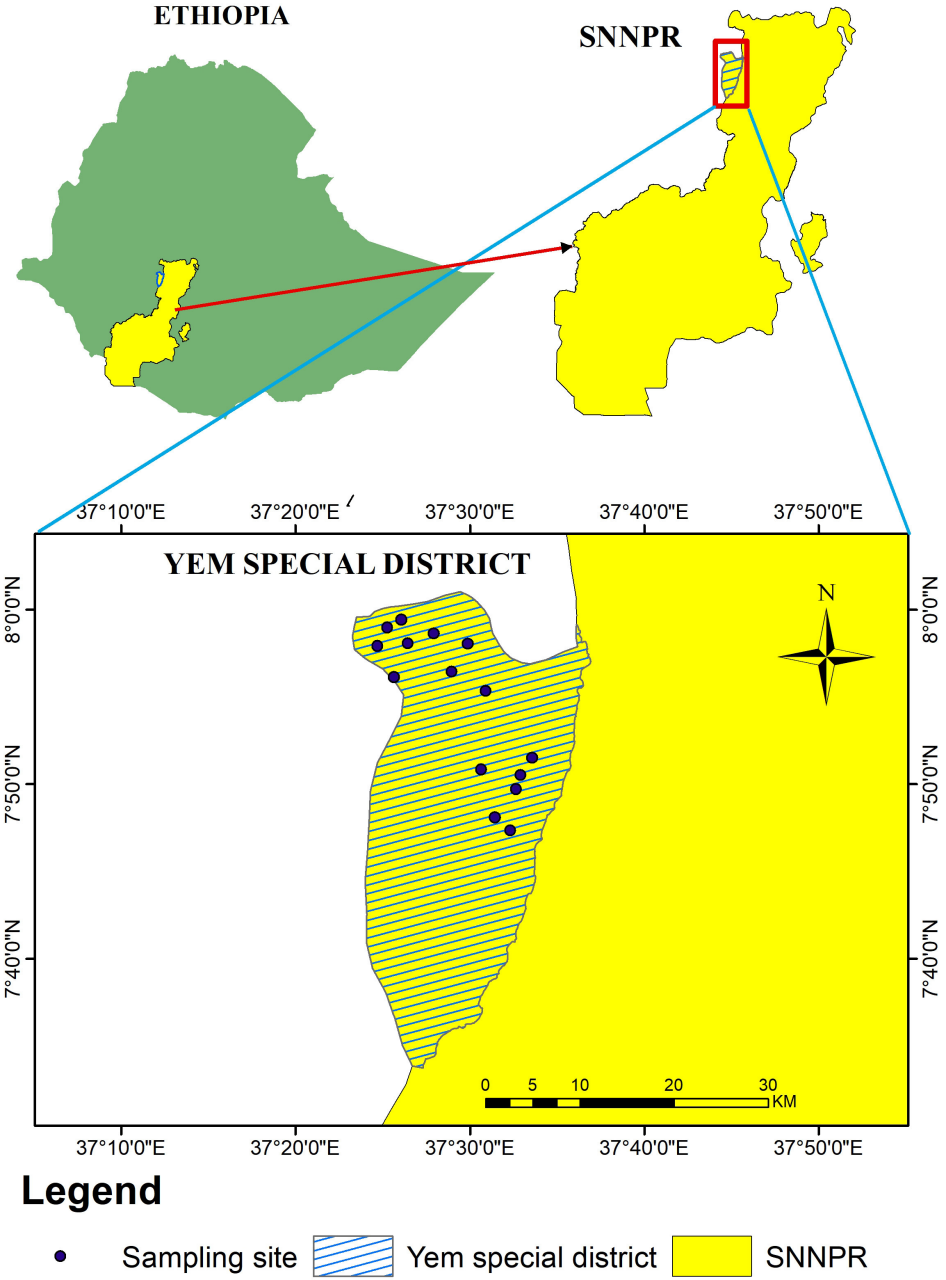
Map of the study area: data is collected from the Yem special district in the Southern Nations, Nationalities, and Peoples' Region (SNNPR) of Ethiopia.

The study area was located in the Southern Nations, Nationalities, and Peoples' Region (SNNPR) of Ethiopia, Yem special district, positioned between latitudes 7^0^57′N and 8^0^02′N, and longitudes 37^0^40′E to 37^0^61′E, covering a total area of 724.5 km^2^, which is ~0.65% of the region. It shares borders with Hadiya and Gurage to the east and south, and Jima (Oromia Region) to the north and west, with the Gibe River delineating its boundary with Hadiya [Figure 1; (11)].

The district lies within elevations of 920–2,939 m above sea level (masl) and has three traditional agroclimatic zones; namely, *Dega* (cool highlands) (18.4%), *Weyna Dega* (tropical highlands) (57.6%), and *Kolla* (lowlands) (24.0%). It receives a mean annual rainfall of 900–2,200 mm in a bimodal pattern, from mid-February to April, and June to September. The mean annual temperature is in the range of 12–30°C (12).

The district's total human population was estimated to be 230,236 of which 50.3% are male and 49.7% female (13), and the population density is 111.3 persons/km^2^. Rain-fed agriculture is the mainstay of the district, the dominant crops being cereals and *enset*. *Enset* is the main staple; the full set of annual field crops cultivated include wheat, barley, *teff* , maize, sorghum, and pulses (14).

The study was conducted in seven peasant associations (PAs), namely Saja Laphto, Ashe, Dicha, Meleka, Oya Kebo, Kelechi, and Deso. There are river basins that flow throughout the year from the district to the Ghibe River system, namely the Kosho River, Zabe River, and Doma River other seasonal rivers that are tributers of the Kosho and Doma are also found.

### Study animals

The study focused on local Zebu cattle. The study area livestock population of bovine is 116,116 (15). The study involved 960 local breed cattle from seven peasant associations in a district (Table 2). The animals were kept under traditional husbandry conditions, grazing communally owned pasture land. Factors such as origin, sex, age, coat color, and body condition score were used to determine the prevalence rate of the animals.

Age was divided into two categories: young (1–3 years) and adult (>3 years) (16). This classification is determined through the examination of teeth, where the wear patterns on molars and premolars indicate whether an animal falls into the young or adult category. Body condition scores were assessed according to guidelines by Nicholson and Butterworth (17), with animals classified as good, medium, or poor, depending on the visibility of ribs and dorsal spines. This method provided a detailed assessment of the health and nutritional status of the cattle, offering valuable insights into their overall wellbeing within the specific management and regional context.

Skin coat colors were identified as white, red, black, and mixed, based on visual observations during the sample collection process, as outlined by Tulu and Diriba (18). This comprehensive approach to classification allowed for a nuanced understanding of the study animals' demographics and health, facilitating targeted analysis and interpretation of the research findings.

### Study design

A cross-sectional survey was conducted from May 2022 to January 2023 to estimate the prevalence of bovine trypanosomes in the Yem special district. The study focused on seven specific peasant associations (PAs), chosen due to their proximity and the prevalence of cattle herding in the area. The sample size was determined for each of the selected PAs based on our total sample size proportional to the number of cattle in that PA. At each PA, the study animals were selected by systematic random sampling from the cattle herds grazing in communal pasture lands. Cattle herds found in PA were the smallest sampling unit in this study. As it was an extensive production system, a herd was defined as those cattle grazing on the same communal pasture. This approach ensured that the sample was representative of the entire population within each PA, minimizing biases that could arise from selective sampling and allowing for a more accurate assessment of trypanosome prevalence across different demographics. Collaboration with local communities and organizations facilitated awareness about the study's objectives, its importance for livestock health, and access to the cattle herds.

### Sample size determination

The sampling size was determined using a formula from Thrusfield and Christley (19), assuming a 50% expected prevalence, a 95% confidence interval, and a desired absolute precision of 5%. Based on the expected prevalence the calculated sample size was 384 animals. However, we collected a larger sample of 960 animals to enhance the precision of our estimates and allow for detailed subgroup analyses within the population.

The choice of a 50% prevalence rate for sample size calculation in our studies was based on statistical principles rather than specific local data. This approach provides the maximum sample size needed to achieve a given confidence level and precision, and ensures that the study is adequately powered to detect the true prevalence, even if the actual prevalence turns out to be lower or higher than 50%. So, in our study area specifically the expected prevalence is unknown, the methods help to maximize the statistical reliability of the findings and ensure the study was able to generate meaningful insights to guide the necessary control measures.

#### Blood sampling and examination

Blood samples were collected from the study animals using heparinized capillary tubes, which were subsequently sealed at one end with a crista seal. These tubes were then centrifuged at 12,000 rpm for 5 min, allowing for the separation of blood components. Following centrifugation, the packed cell volume (PCV) was measured to assess the blood's hematocrit level, as documented by Samdi et al. (20). A PCV value below 24% was used as the threshold for diagnosing anemia, based on criteria set forth by Van den Bossche and Rowlands (21).

For the detection of trypanosomes, the buffy coat, along with the uppermost layer of red blood cells from each sample, was transferred onto a microscope slide for examination, a method outlined by Murray et al. (22). In cases where blood samples tested positive for trypanosomes, thin blood smears were prepared, stained with Giemsa stain, and scrutinized under light microscopy for species identification. The differentiation of Trypanosoma species was conducted by analyzing morphological features such as size, the position of the kinetoplast, the extent of undulating membrane development, and the presence or absence of a free flagellum, following the guidelines provided by Desquesnes (23). This systematic approach enabled not only the detection of trypanosomes but also the identification of the specific Trypanosoma species involved, thereby facilitating a more targeted understanding and management of the disease.

#### Entomological study

In the Yem special district, 86 traps were strategically set within selected peasant associations (PAs) known for prevalent tsetse fly activity, targeting environments like gallery forests, riverbanks, and regions frequented by wild game. These traps were baited with acetone, octanol, and cow urine to lure tsetse flies, as Brightwell et al. (24) recommended. To optimize their effectiveness, the traps were spaced ~200–250 m apart and surrounded by a cleared radius of 2–3 m to both enhance visibility and minimize fire risks. Each trap remained in place for 72 h, providing ample opportunity for attracting and capturing tsetse flies.

Upon collection, the tsetse flies were meticulously identified to species or genus level based on distinct morphological traits, adhering to the guidelines set by the Food and Agriculture Organization (25). This process of sorting, counting, and identifying by species was conducted (26). Additionally, the precise locations of the traps were documented using GPS technology to ensure accurate geographical referencing.

The data analysis focused on the number of flies caught and their specific species composition, facilitating the calculation of tsetse flies' apparent density at each trap location. The apparent density was quantified as the average number of flies captured per trap per day (FTD), a method validated by Leak (27).

### Data analysis

During the research, data collected were systematically organized and stored in Microsoft Excel^®^ Windows 2010, providing a structured and accessible format for analysis. The analysis was conducted using STATA version 14.2 for Windows, a statistical software package from Stata Corp., College Station, TX, USA.

To calculate the prevalence of trypanosomes, the study employed a straightforward method where the number of positive cases (samples testing positive for the parasite) was divided by the total number of samples collected.

Further, the mean Packed Cell Volume (PCV) values were compared with infected and uninfected cattle populations. A *t*-test was used for this comparison, enabling the determination of any statistically significant differences in the PCV values, which are indicative of the health status of the cattle, particularly concerning anemia associated with trypanosomes.

Statistical significance was determined at the 0.05 probability level with a 95% confidence interval (CI), ensuring that the observed differences or associations were unlikely to have occurred by chance. The Chi-square test was used to identify significant risk factors associated with trypanosomiasis in cattle. This threshold for significance is a standard criterion in scientific research, providing a balance between sensitivity to detect real effects and the control of false-positive rates.

For the entomological component of the study, the apparent density of tsetse flies was calculated. This was achieved by dividing the total number of tsetse flies captured (∑F) by the product of the number of working traps (T) and the number of days these traps were operational (D). This metric, expressed as the number of fly catches per trap per day, offers a quantitative measure of the tsetse fly abundance in the study area, crucial for assessing the risk of trypanosome transmission and guiding vector control strategies.

## Results

### Parasitological study results

In the parasitological component of this study, a total of 960 cattle were examined for Trypanosoma parasites, with 102 animals testing positive. This resulted in an overall trypanosome prevalence of 10.6%(9–12%) across the study areas. Notably, the highest prevalence rates were observed in the Decha and Saja Laphto peasant associations, although no statistical difference was found between the various associations investigated (Table 1).

**Table 1:** The overall prevalence of bovine trypanosomes in different PAs of Yem special district.

**PA**	**No. of the examined animals**	**Infected animals**	***Trypanosoma*** **spp**.			**Prevalence % (95% CI)**	***X* ^2^**	***P*-value**
			**T.c**	**T.v**	**Mixed**			
Ashe	200	19	11	7	1	9.5 (6–13)	5.37	0.497
Deso	53	8	2	5	1	15.1 (7–27)		
Decha	200	23	11	11	1	11.5 (8–17)		
Meleka	200	16	12	3	1	8 (5–12)		
Oya kebo	100	9	4	5	0	9 (4–17)		
Saja laphto	147	21	12	8	1	14.3 (9–23)		
Kelechi	60	6	4	1	1	10 (3–19)		
Total	960	102	56	40	6	10.6 (9–12)		

T.c, Trypanosoma congolense; T.v, Trypanosoma vivax; X^2^, Chi-square; Spp, Species.

The study also explored several hypothesized risk factors for trypanosomes. A significant difference in prevalence was identified based on the sex of the animals, with males exhibiting a higher prevalence compared to females, and this difference was statistically significant (*p* < 0.001). Age also proved to be a significant risk factor, with adult cattle showing a higher prevalence than younger animals, indicating a significant variation.

When considering the color of the animals, the study found that black cattle had the highest prevalence of trypanosomes compared to white and red animals. However, this difference was not statistically significant (*p* = 0.119). Similarly, cattle in poor body condition showed a higher prevalence of trypanosomes compared to those in good and medium body condition animals, but again, the difference did not reach statistical significance (Table 2).

**Table 2:** Prevalence of trypanosomes in relation to body condition, age, sex, and coat color of the animals.

**Risk factors**	**Number of animals examined**	**Infected animals**	**Prevalence %**	***X* ^2^**	***P*-value**
**Body condition**					
Good	195	13	6.7	5.36	0.068
Medium	454	41	9.03		
Poor	311	48	15.43		
**Age**					
Young ( ≤ 3 years)	262	18	6.9	5.35	0.021
Adult (>3 years)	698	84	12		
**Sex**					
Male	577	79	13.7	14.32	0.000
Female	383	23	6		
**Hair coat color**					
White	57	7	12.3	5.86	0.119
Black	127	74	58.3		
Red	768	21	2.7		
Gray	8	0	0		

X^2^, Chi-square.

#### Species of trypanosomosis identified

Two species of trypanosomes, *Trypanosoma congolense*, and *Trypanosoma vivax*, were identified in Yem special district. *Trypanosoma congolense, Trypanosoma vivax*, and mixed infection with both *Trypanosoma congolense* and *Trypanosoma vivax* were confirmed during the investigation. In this study area, *T. congolense* was the predominant species of the overall infection.

#### Hematological finding

In the study of 960 cattle, the Packed Cell Volume (PCV) values exhibited a distribution with an average PCV of 22.80% and a standard deviation of 4.85%. Among these, the most common PCV value was 22%, observed in 128 animals. On the lower end of the frequency distribution, nine animals exhibited the least frequent PCV values within the study group. For animals that tested positive for trypanosomes, the highest PCV noted was 28%, and the lowest was 10%. The analysis revealed a significant difference in the mean PCV values between infected and non-infected animals. Infected animals had a lower mean PCV of 18.94% (± 3.92SD) compared to 23.26% (± 4.74SD) observed in non-infected animals, indicating a statistically significant reduction (*P* < 0.001) in the PCV among the infected group (Table 3).

**Table 3:** The mean packed cell volume (PCV) of examined cattle in the Yem special zone.

**Infection status**	**Number of animals**	**Mean PCV**	**SE**	**SD**	**95% CI**	***t*-test**	***P*-value**
Non-infected	858	23.26	0.16	4.74	22.94–23.58		
Infected	102	18.94	0.39	3.92	18.17–19.71	8.8456	< 0.001
Total	960	22.80	0.16	4.85	22.50–23.11		

PCV, Packed cell volume; SE, Standard error; SD, Standard deviation; CI, Coefficient interval.

### Entomological study results

During the study period, a total of 565 tsetse flies were captured using 86 traps deployed in the selected peasant associations within the Yem special zone. Among the captured flies, three species of Glossina were identified: *G. f. fuscipes, G. pallidipes*, and *G. morsitans submorsitans*. The distribution of these species varied, with *G. morsitans submorsitans* being the most prevalent followed by *G. pallidipes* and *G. f. fuscipes*.

**Figure 2:**
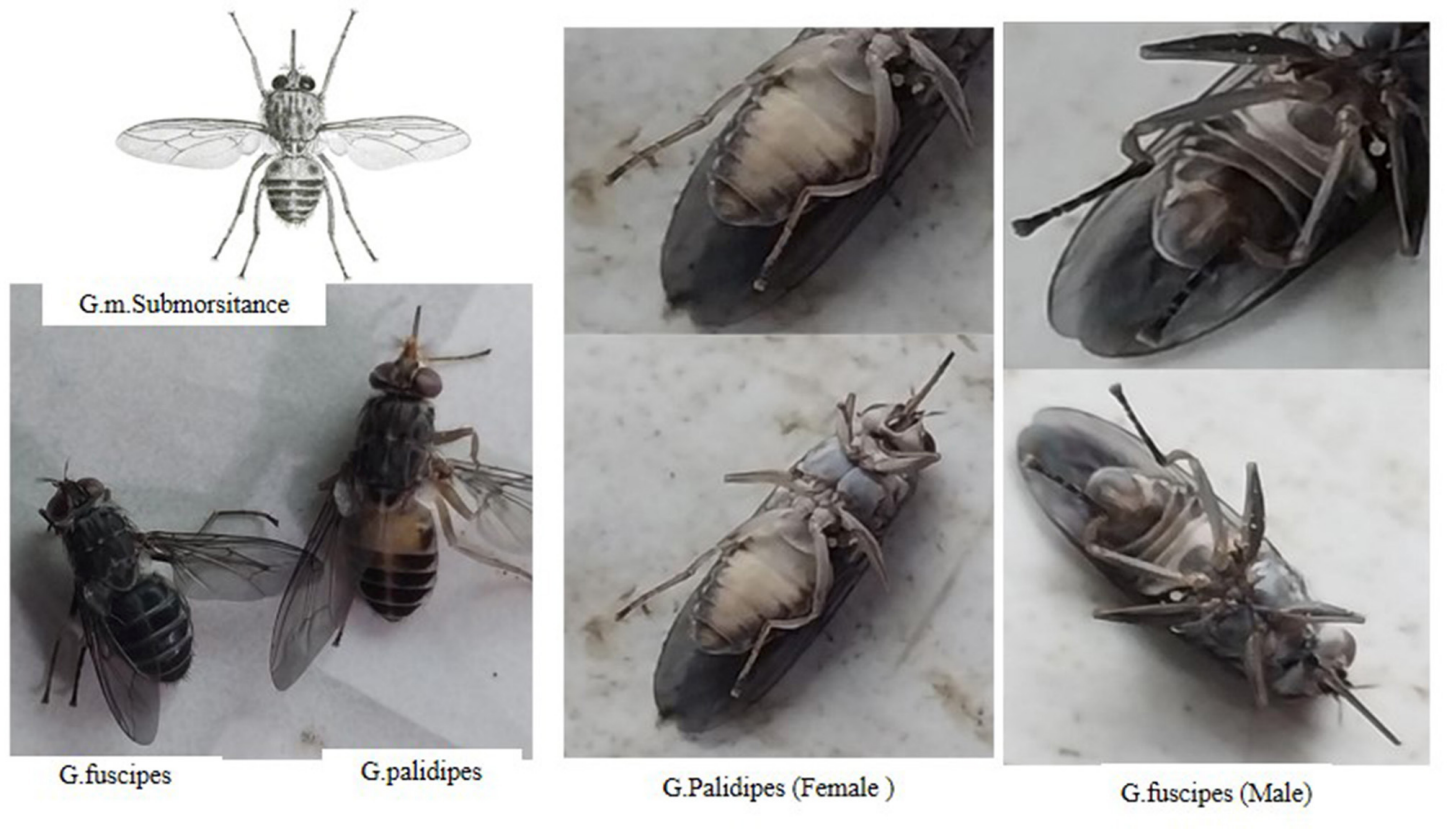
Three species of tsetse fly identified in the study area.

The overall apparent density of the Glossina species was calculated to be 2.19 flies per trap per day (FTD). When broken down by species, the apparent densities were 0.91 flies per trap per day for *G. morsitans submorsitans*, 0.58 for *G. f. fuscipes*, and 0.69 for *G. pallidipes* (Table 4). These figures illustrate the relative abundance and distribution of each tsetse fly species in the study areas, providing crucial data for understanding the potential vector dynamics and transmission risks of trypanosomes in the Yem special zone (Figure 2).

**Table 4:** Apparent density of flies trapped in three days in different PAs in Yem special district.

**PA**	**Number deployed of traps**	**Glossina species**			**Total**	**FTD**
		***G. morsitans***	***G. f. fuscipes***	***G. pallidipes***		
Ashe	17	8	88	28	124	2.43
Decha	27	130	12	68	210	2.59
Meleka	21	79		20	99	1.57
Oya Kebo	21	19	50	63	132	2.10
Total	86	236 (41.77%)	150 (26.55%)	179 (31.68%)	565	2.19

PA, Peasant associations; FTD, Fly per trap per day.

## Discussion

This parasitological investigation within the Yem special district underscores the widespread nature of bovine trypanosomes, validating its status as a significant hindrance to cattle farming, in line with previous findings (2, 28, 29). The recorded overall trypanosome prevalence of 10.6% aligns closely with similar research conducted in various regions of Ethiopia, which reported prevalence of 9.6, 10.1, and 10.05%, respectively (2, 30, 31). However, this figure is somewhat lower than other reports from the Wolyta and Dawero Zone (20.4%), Sayo District (16.9%), Botor Tolay District (12.24%), Metekel and Awi zones (12.41%), and significantly lower than figures reported from Kenya (41 and 29%) (6, 32–34). Conversely, it surpasses prevalence rates observed in the Didesa district (4.86%), Arbaminch (4.43%), Lalo Kile district (7.78%), Darimu district (7.1%), and Quara district (6.77%) (4, 35, 36).

Variations in prevalence might stem from differential agroecological conditions, vector control initiatives, diagnostic methodologies, and cattle management practices across these areas. Furthermore, this study emphasizes the necessity for area-specific control measures by exploring socio-economic factors affecting disease transmission, including human-wildlife interactions, land use practices, and access to veterinary care. Enhanced community-based surveillance and sustainable land management strategies are vital for mitigating trypanosomes and other vector-borne diseases. Overall results align with the findings of Degneh et al. and Mekuria and Gadissa (28, 30), who reported similar rates in regions with comparable ecological conditions. However, higher prevalence rates were observed in areas such as Wolyta and Dawero zones, which provide more favorable environments for tsetse flies that thrive in dense vegetation and warm climates (32). Regions that implement vector control measures often report lower infection rates due to reduced exposure to vectors (34), while studies using more sensitive diagnostic techniques may detect lower levels of infection compared to those employing less rigorous methods (6).

Notable differences in husbandry practices, sampling seasons, vector control efforts, and environmental changes due to deforestation could explain the disparity in prevalence rates within Ethiopia, suggesting a need for a nuanced approach to disease management and control (37). Our study identified the highest prevalence in Decha and Saja Laphto, attributing variances to ecological conditions, vector prevalence, and trypanocidal drug use, emphasizing the impact of these factors on disease epidemiology (37, 38).

Pathogenically, two important pathogenic trypanosome species of cattle, *T. congolense* and *T. vivax* were identified at a prevalence of 56 (5.83%) and 40 (4.17%), respectively, which agreed with the observations made by other groups in different tsetse infested areas in Ethiopia (62, 79). The highest prevalence due to *T. congolense* in this study may suggest an increased presence of the biological vectors for *T. congolense* and reduced occurrence of other biting flies, which have been implicated in the mechanical transmission of *T. vivax* (39). It could be because of a better immune response to *T. vivax* by the infected animals (40). According to Stephen and Van den Bossche and Rowlands (21, 41), *T. congolense* is mainly confirmed in the blood, while *T. vivax* and *T. brucei* also invade the tissues. The higher proportion of *T. congolense* in the current study was similar to the previous results (4, 28, 30, 42), because of the environmental conditions that support tsetse fly habitats, such as specific temperatures, humidity, vegetation types, and host availability may result in similar patterns of trypanosomes species distribution.

*T. congolense's* prevalence in cattle, particularly in sub-Saharan Africa, underlines the chronic nature of these infections. Infected animals may carry the parasite for an extended period, leading to a higher prevalence over time. Chronic infections can also contribute to the maintenance of *T. congolense* in the cattle population, necessitating improved livestock health management, understanding host-parasite dynamics, and developing innovative control measures (38, 43, 44).

Our, findings on body condition and trypanosomes prevalence highlight the disease's debilitating effects, with poor-condition animals showing higher infection rates, which has similar to the previous reports (31, 45–48). It is well-known that trypanosomosis is a wasting disease that leads to a progressive loss of body condition (49), although other factors such as parasitism and nutritional stress may also play a role in the development of poor body condition (78). Conversely, poor body condition can also be a risk factor for trypanosomosis, as animals with poor body condition have weaker immunity and are therefore at higher risk of infection with trypanosomes. This interplay suggests that improving nutritional and health management could bolster resistance against trypanosomes (45, 48).

The findings of our study indicated a higher prevalence of trypanosomes in black-color animals compared to white and red animals. This variation can be attributed to the feeding behavior of tsetse flies, which are more attracted to darker-colored animals due to their coloration resembling the natural hosts of these vectors. This enhanced attraction may result in increased exposure and subsequent infection rates among black cattle compared to white and red-color animals (50, 51).

Age related susceptibility patterns were showed that there was a significant difference (*P* < 0.05) in prevalence between the age groups. A higher infection rate of 84 (8.75%) was observed in adult animals (>3 years) and a lower infection rate (1.88%) in young animals (<3 years). This could be related to older animals in tsetse-prone areas traveling long distances to graze, move, and harvest crops (4, 5). Adult cattle often exhibit a higher social status, which may lead them to engage in activities that heighten their risk of exposure to tsetse flies (52). Additionally, the larger body size of adult cattle may make them more attractive as hosts for tsetse flies, further increasing their likelihood of infection (53). The habitats preferred by adult cattle, such as humid environments with abundant vegetation, often overlap with the ecological niches inhabited by tsetse flies, heightening the chances of contact between infected tsetse flies and the adult cattle population. This increased exposure potentially explains the higher prevalence of trypanosomes among adult cattle in the study region (54, 55). However, young animals are naturally protected to a certain extent by maternal antibodies, and tsetse flies are significantly more attracted to the odor of older animals and animals that show less defensive behavior (56). Similar observations were made by Degneh et al. (28), Terefe et al. (57), Torr et al. (58), and Tulu and Diriba (18) in Ethiopia, where they reported the effect of age on the prevalence of trypanosome infections. Furthermore, *T. congolense* is a chronic disease that increases with the animal's age, and infection is usually higher in adult animals than in young animals (59, 74).

In the current study, the prevalence of bovine trypanosomosis was significantly different between the sexes (*P* < 0.05), notably higher in male animals than females. Different Authors also reported higher infection rates in male animals than females (60–63) indicating higher prevalence in male cattle than in females. The higher infection rate in males may be attributed to stress factors related to work where the animals are used for drought and they have to travel long distances in areas where there is a high risk of tsetse infection (64). Additionally, management related risk factors are that male animals are mostly managed in extensive grazing fields where there is a high risk of tsetse challenge for longer hours than the female ones which are managed around the house for milk yield, especially during the rainy season, which facilitates exposure to fly bites and thereby the chance of getting infected by Trypanosomes (65). On the other hand, reports in Ethiopia (28, 36, 62), and Nigeria (66, 67) showed a significantly highest prevalence of trypanosomes in female animals than male, suggested that the relatively higher prevalence observed in females the stress associated with hormonal imbalances during pregnancy and lactation, which usually increases females' susceptibility to infections (58).

Lastly, the study's entomological findings, revealing an apparent density of 2.19 flies/trap/day, offer a critical metric for assessing and strategizing vector control efforts. These findings were lower than the previous report 14.97 f/t/d from Arbaminch (36) and 11.9 f/t/d from HewaGelan district, Oromia region, west Ethiopia (68). The current result was also higher than the previous report 1.15 f/t/d for tsetse in the East Wollega zone (69), 2.83 f/t/d from the Bench Maji zone (70), and 1.35 f/t/d in southern rift valley of Ethiopia (71). Their specific habitat preferences influence the distribution of tsetse fly species. For instance, *G. morsitans submorsitans* is commonly found in savannah and woodland areas, which may be more prevalent in the study areas compared to the habitats favored by *G. pallidipes* and *G. f. fuscipes*, which thrive in more humid environments such as riverbanks and forested areas (55). The density of potential hosts can significantly impact the population dynamics of tsetse flies. Areas with higher cattle populations support larger populations of *G. morsitans submorsitans*, as this species is known to have a strong association with livestock (72). Additionally, seasonal changes affect fly activity and density, influencing trap catches. For instance, during the rainy season, certain species may become more active or migrate to different areas, affecting their prevalence in traps set at specific times (73).

In our study, other biting flies were not captured. The physical design and dimensions of the traps specifically target tsetse flies, which are not suitable for effectively capturing larger or more aggressive biting fly species, which could have also contributed to their absence from the collected samples. The attractants utilized were particularly appealing to tsetse flies, but their efficacy was limited for other types of biting flies. Additionally, the strategic positioning of traps in areas known for tsetse fly prevalence also has restricted capture to other biting fly species.

Recognizing the variability in tsetse fly populations and their implications for disease transmission is essential for developing effective trypanosomes control measures, emphasizing the importance of continuous research and adaptive management strategies to address this enduring challenge.

## Conclusion and recommendation

This study conclusively demonstrates a significant prevalence of 10.6% (0.09–0.12) trypanosomes in cattle, highlighting its detrimental impact on livestock health and agricultural productivity in the surveyed regions. *Trypanosoma congolense* and *Trypanosoma vivax* emerge as primary pathogens, with age and sex identified as notable risk factors exacerbating the disease's spread. The predominance of *G. morsitans submorsitans* among tsetse fly vectors underscores the urgent need for targeted vector control measures.

Addressing this challenge requires an integrated management strategy, emphasizing not only the use of insecticide-treated interventions but also habitat modification and livestock management practices to mitigate tsetse fly contact. Crucially, the engagement and education of local communities play a pivotal role in enhancing disease surveillance, prevention, and control efforts. Further research, particularly molecular techniques, is essential for a deeper understanding of disease mechanisms and developing innovative control solutions.

## Data Availability

The original contributions presented in the study are included in the article/supplementary material, further inquiries can be directed to the corresponding author.
